# A nested case-control study of circular ribonucleic acid expression profiles in the peripheral blood of pregnant women with pre-eclampsia

**DOI:** 10.12669/pjms.40.11.10317

**Published:** 2024-12

**Authors:** Qiuping Liao, Lin Zheng, Liangpu Xu, Lingling Jiang, Jinying Luo, Jianying Yan

**Affiliations:** 1 Qiuping Liao, Gynaecology and Obstetrics, Fujian Maternity and Child Health Hospital, College of Clinical Medicine for Obstetrics & Gynecology and Pediatrics, Fujian Medical University, Fuzhou, Fujian Province 350001, China; 2 Lin Zheng, Medical Genetic Clinic, Fujian Maternity and Child Health Hospital, Fuzhou, Fujian Province 350001, China; 3 Liangpu Xu, Medical Genetic Clinic, Fujian Maternity and Child Health Hospital, Fuzhou, Fujian Province 350001, China; 4 Lingling Jiang, Gynaecology and Obstetrics, Fujian Maternity and Child Health Hospital, College of Clinical Medicine for Obstetrics & Gynecology and Pediatrics, Fujian Medical University, Fuzhou, Fujian Province 350001, China; 5 Jinying Luo, Gynaecology and Obstetrics, Fujian Maternity and Child Health Hospital, College of Clinical Medicine for Obstetrics & Gynecology and Pediatrics, Fujian Medical University, Fuzhou, Fujian Province 350001, China; 6 Jianying Yan, Gynaecology and Obstetrics, Fujian Maternity and Child Health Hospital, College of Clinical Medicine for Obstetrics & Gynecology and Pediatrics, Fujian Medical University, Fuzhou, Fujian Province 350001, China

**Keywords:** Pre-eclampsia, Circular ribonucleic acid, Gene expression profiling, Biomarkers

## Abstract

**Objective:**

To examine the expression of circular ribonucleic acid (circRNA) in the peripheral blood of pregnant women with preeclampsia (PE) prior to the onset of the disease.

**Method:**

A prospective nested case-control study included pregnant women who delivered at Fujian Maternal and Child Health Hospitals between November 2021 and October 2022. The study included three women diagnosed with PE (the PE group) and three healthy pregnant women (the control group). The expression of circRNAs in the peripheral blood before clinical diagnosis at 20 weeks gestation was analyzed by microarray technology, and the functional analysis and regulatory network prediction were done.

**Result:**

There were 162 circRNAs with differentially expressed multiplicity of > 2.0 in the PE group compared to the control group. Out of them, 30 circRNAs were up-regulated and 132 were down-regulated. Gene ontology (GO) analysis revealed that the host genes of the differentially expressed circRNAs mainly participated in protein localization to plasma membrane, insemination, etc. Most enriched terms were related to cytoplasmic dynein complex, microtubule cytoskeleton, etc. In terms of molecular function, the enriched terms were related to dynein light intermediate chain binding, polyubiquitin modification-dependent protein binding, etc. Kyoto Encyclopedia of Genes and Genomes (KEGG) signaling pathway enrichment analysis revealed that differentially expressed circRNAs were involved in vasopressin-regulated water reabsorption, cyclic guanosine monophosphate-protein kinase G (cGMP-PKG), calcium- and other signaling pathways.

**Conclusion:**

CircRNAs are involved in a variety of biological processes and regulatory network related to the pathogenesis of PE.

## INTRODUCTION

Pre-eclampsia (PE) is an obstetric syndrome that occurs in the middle and late stages of pregnancy, and is characterized by elevated blood pressure after 20 weeks of gestation, accompanied by at least one complicating symptom, such as proteinuria, maternal organ damage or uteroplacental dysfunction. PE is considered an important cause of maternal and perinatal fetal death^1^ that affects four million pregnancies and result in the deaths of more than 70,000 women and 500,000 newborns worldwide each year.^2^ Studies have suggested that abnormal placental trophoblast function, including the proliferation of placental trophoblasts, the lack of infiltration capacity and abnormal remodeling of the uterine spiral arteries, plays a key role in the pathogenesis of PE. However, the exact molecular mechanisms have not yet been clarified.^3^

Current advances in transcriptomics research and the development of RNA sequencing technology led to detection of numerous non-coding RNAs. Recent studies have demonstrated an important correlation of non-coding RNAs with the pathogenesis of severe PE, and showed that epigenomic regulation may play an important role in the pathogenesis of hypertension.^4^ Circular RNAs (circRNAs) are newly discovered endogenous non-coding RNAs that can regulate genes at the post-transcriptional level.^5^ They participate in many signaling pathways that play important roles in human diseases, making circRNAs promising potential biomarkers for human diseases.^6^ Current studies have reported high levels of circRNA in both peripheral blood and placental tissue of pregnant women.^7^,^8^ However, little is known about the ability of circRNA to serve as an early predictor of PE before its onset. At present, there is no clear and effective clinical treatment measure for PE. Therefore, the prediction of PE risk in early pregnancy and the targeted management of high-risk pregnant women are crucial. In this paper, we examined the expression of circRNA in the peripheral blood of pregnant women with PE before the onset of the condition, with the aim of exploring a new means of early PE prediction. Our results may provide a new theoretical basis for PE prevention and treatment.

## METHOD

This nested prospective case-control study which included pregnant women who underwent regular antenatal checkups and gave birth in Fujian Maternal and Child Health Hospitals between November 2021 and October 2022. The first antenatal checkup was taken as the starting point of the cohort, and the information of the first antenatal checkup was taken as the baseline data. Routine antenatal checkups were taken as the time of follow-up until the delivery. Pregnant women diagnosed with PE (n=3) were included in the PE group. Pregnant women without PE (n=3) were selected as the control group in the ratio of 1:1, with a difference in gestational weeks of less than seven days, a difference in the number of previous pregnancies of less than two, and the same number of previous deliveries at the time of collecting blood samples.

PE was diagnosed in accordance with the Chinese Expert Consensus Criteria of the Guidelines for the Diagnosis and Treatment of Hypertensive Diseases in Pregnancy (2020).^9^ Exclusion criteria were as follows: advanced age, gynecological disorders in combination with pregnancy, trophoblastic diseases, liver, kidney, heart, lungs, and hematological diseases, metabolic disorders in the pre-pregnancy period, and non-single pregnancies.

### Ethical Approval:

This study was approved by the ethics committee of Fujian Maternity and Child Health Hospital (FMCH2020-2014), Date: March 26^th^ 2020. Informed consent was obtained from all subjects involved in the study.

Before 20 weeks of gestation, a peripheral whole blood (3ml) sample was collected and stored at -80 °C. Total RNA was extracted using the TRIzol ® kit (Invitrogen, Life technologies, USA) according to the manufacturer’s instructions. NanoDrop ®ND-1000 (Agilent, USA) was used to determine the concentration and purity of RNA based on the OD260/OD280 ratio. RNA integrity was determined by denaturing agarose gel electrophoresis. Arraystar Super RNA Labeling Kit (Arraystar, USA) was used to label circRNA with the labelling enzyme Hy3 fluorophore to obtain fluorescent probes for microarray hybridization. Human Circular RNA Array Kit (containing 5,396 circRNAs) from Arraystar, USA was used to generate circRNA expression microarray slides. The slides were incubated for 17 h and hybridized in the hybridization oven (Agilent, USA) at 65 °C.

After hybridization and fixation, the microarray slides were scanned using Axon GenePix 4000B chip scanner (MolecularDevices, USA). GenePix Pro 6.0 software (Axon, USA) was used to analyze the raw data. Data were normalized using the R package, and differentially expressed multiplicity >2.0 was the differentially expressed circRNA. Gene ontology (GO) analysis, Kyoto Encyclopedia of Genes and Genomes (KEGG) pathway database, and CircNet database were used for the analysis. GO Analysis (http://www.geneontology.org/) is a database describing gene products, providing attributes that define gene-related products. The GO number of the differential gene allows to find GO classification entries that are enriched for differential genes and to look for the relationship between the differential gene and altered gene function.

The KEGG pathway database (http://www.genome.jp/kegg/pathway.html) is an online set of databases on genomes, enzymatic pathways, and biochemicals. Differential genes allow to find pathway entries enriched for differential genes. The CircNet database (http://circnet.mbc.nctu.edu.tw/) for endogenous RNA function studies was applied to predict circRNA-miRNA-mRNA regulatory networks.

### Statistical analysis:

SPSS 20.0 statistical software package was applied for statistical analysis. Normally distributed measurements were expressed as *χ̅*±S, and t-test was used for comparison between groups. A two-sided test was used with a test level of α = 0.05.

## RESULTS

There was no significant difference in the age at delivery (p = 0.854), pre-pregnancy BMI (p= 0.911, and pre-partum BMI (p= 0.677) between the PE and the control groups (Table-I). The gestational week of delivery in the PE group was significantly lower than that in the control group (p < 0.05), and the systolic and diastolic blood pressures in the PE group were significantly higher compared to the control group (p< 0.01).Table-I

**Table-I:** Comparison of basic information and blood pressure of pregnant women in the two groups (*χ̅*±*S*).

Categories	PE group (n=3)	Control group (n=3)	t value	p-Value
Age (years)	29.67±2.52	29.33±1.53	0.196	0.854
Pre-pregnancy BMI (Kg/m2)	21.48±2.11	21.67±1.78	-0.119	0.911
Prenatal BMI (Kg/m2)	28.22±2.91	27.30±2.07	0.448	0.677
Week of gestation at delivery (weeks)	36.13±0.85	39.17±0.90	-4.238	0.013
Systolic blood pressure (mmHg)	151.00±3.60	109.00±3.61	14.267	0.000
Diastolic blood pressure (mmHg)	96.00±4.00	65.67±4.04	9.240	0.001

***Note:*** PE: preeclampsia; BMI: body mass index.

**Fig.1:**
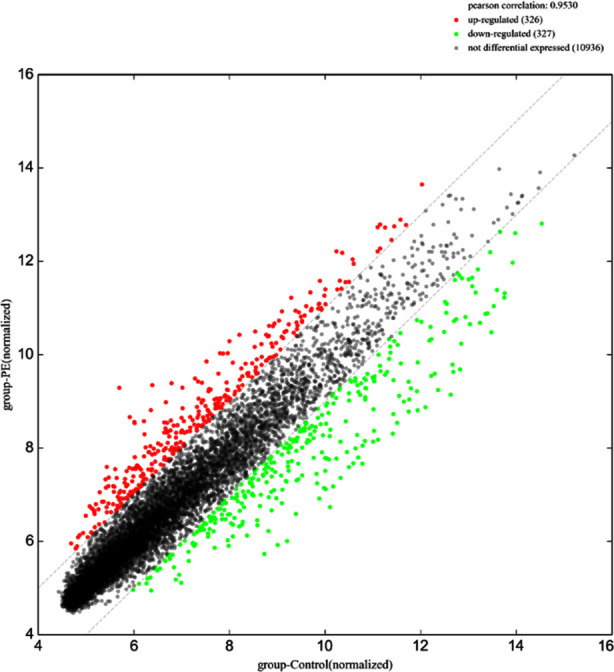
Scatter plot of signal values of circRNA expression in peripheral blood of pregnant women in PE and control groups.

**Fig.2:**
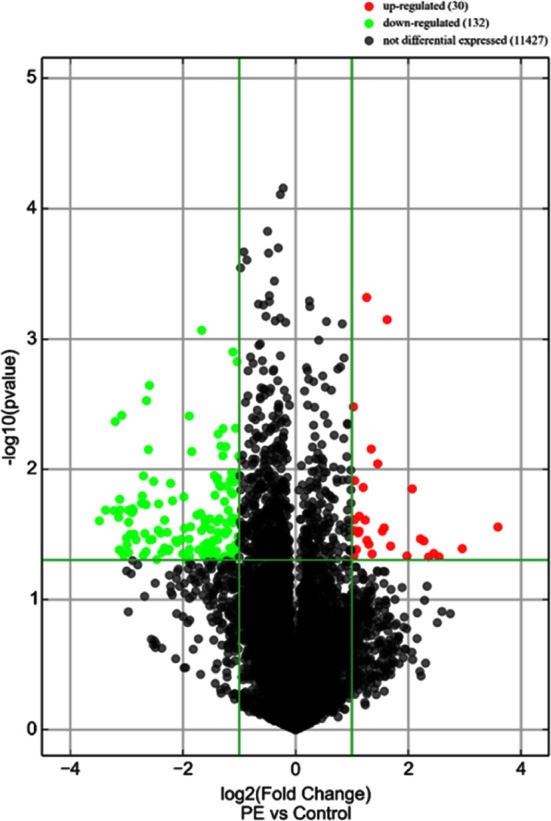
Volcano plot of differentially expressed circRNAs in the peripheral blood of pregnant women in the PE and control groups.

**Fig.3:**
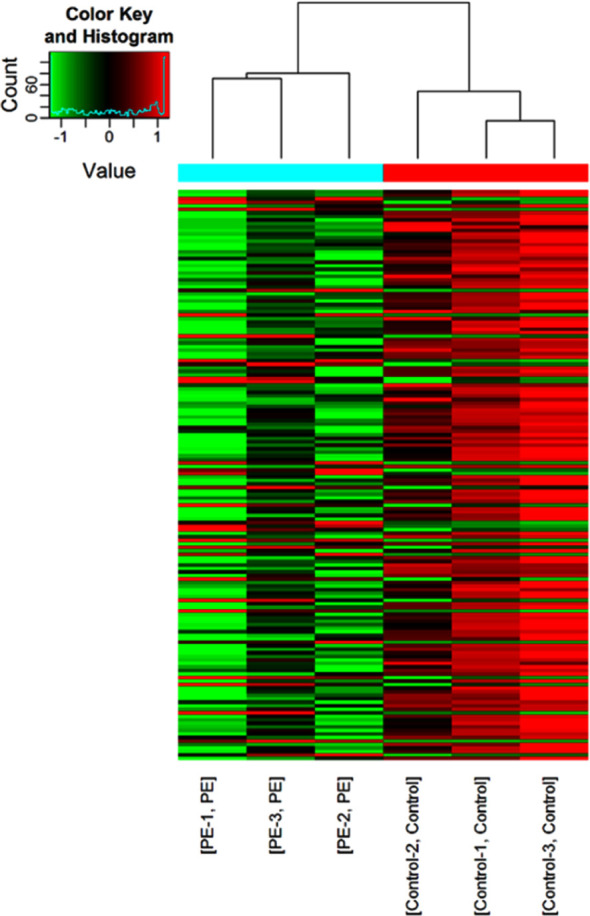
Heatmap of signal values of circRNA expression in the peripheral blood of pregnant women in the PE and control groups and clustering results (red indicates up-regulated expression, green indicates down-regulated expression).

Scatter plot of signal values of circRNA expression in the peripheral blood of pregnant women in the PE group and the control group are shown in Fig.1. The data were then combined with ploidy and p-Value to draw a volcano plot for the screening of differentially expressed circRNAs. A total of 30 differentially expressed circRNAs with a ploidy of >2.0 were up-regulated and the expression of 132 circRNAs was down-regulated (p< 0.05) (Fig.2). To further show the relationship between PE and the differential circRNA expression, the expression signal values of up-regulated and down-regulated circRNAs were plotted as heat maps and the specimens were clustered and analyzed respectively. The results showed that pregnant women with and without PE could be clustered into two groups separately, indicating a greater degree of similarity in circRNA expression signal values between each group of samples, and a smaller degree of similarity, i.e., a greater degree of difference, in circRNA expression signal values between the two groups of pregnant women (Fig.3).

**Fig.4:**
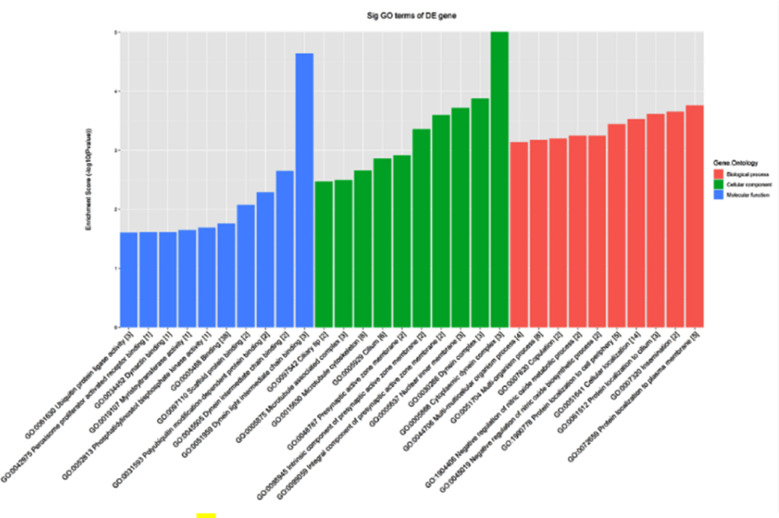
Gene function analysis of differentially expressed circRNAs.

**Fig.5:**
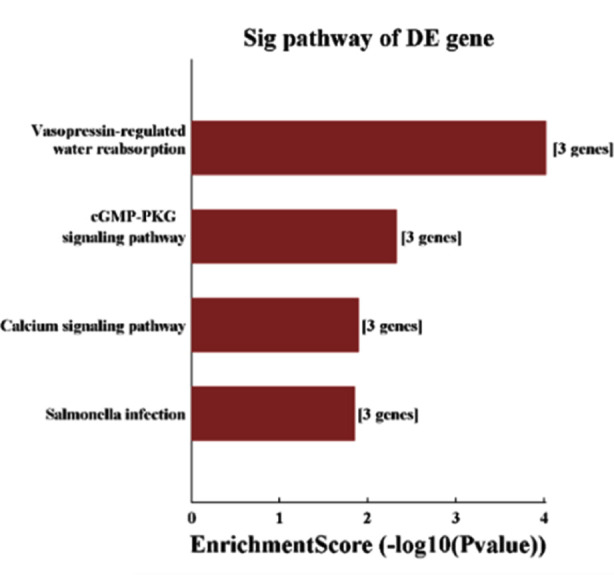
Enrichment analysis of KEGG signaling pathway for differentially expressed circRNA target genes.

Functions of differentially expressed circRNA genes were mined in the GO database (Fig.4). KEGG PATHWAY analysis revealed that circRNA target genes were enriched in signaling pathways including vasopressin-regulated water reabsorption, cGMP-PKG signaling pathway, calcium signaling pathway, etc. (Fig.5).

CircNet database was used for the analysis. Hsa_circ_0065049 was found to affect the expression of 81 target gene mRNAs by binding to 8 miRNAs, including miR-17-3p, miR-1226-5p, miR-3912-5p, miR-4640-5p and miR-4751; hsa_circ_0005567 could affect the expression of 12 target gene mRNAs by binding to 6 miRNAs, including miR-24-3p, miR-1270, miR-4265, miR-4322, and miR-4296.

## DISCUSSION

Our study identified 30 up-regulated and 132 down-regulated circRNAs in the periphery blood of pregnant women. We show that circRNAs are involved in a variety of biological processes and regulatory networks related to the pathogenesis of PE. The regulatory networks that might be related to PE were predicted to be located on hsa_circ_0065049 and hsa_circ_0005567, respectively.

PE is one of the most important causes of maternal and perinatal fetal death, and its complexity makes treatment more difficult.^3^ Since PE produces pathological changes months before the onset of clinical symptoms, prediction and prevention of the disease is crucial, and specific preventive or interventional measures for specific populations are needed.

Current advances in proteomics technology allowed to identify several predictive biomarker molecules for PE. These biomarkers include soluble fms-like tyrosine kinase1, (sFlt-1), placental growth factor (PlGF), soluble endoglin (sEng), activator A, inhibitor A, pregnancy-associated plasma protein A (PAPP-A), lipid peroxides and antioxidants, inflammatory and immune-related factors, coagulation and fibrinolytic-related factors, and microRNAs (miRNAs).^3^,^10^–^14^ However, none of these biomarkers had high predictive efficacy either alone or in combination with additional predictors.^15^ In this study, we used sequencing technology to construct the differential expression profiles of circRNAs in the peripheral blood of pregnant women with PE before the onset of the disease, aiming to explore a new means of early prediction of PE, and at the same time provide a new theoretical basis for the prevention and treatment of PE.

Circular RNAs are intact, conserved, and resistant to the action of RNase R,^6^ and are present in a wide variety of organisms, with differential expression in different tissues or in different diseases.^16^,^17^ More and more studies have shown that circRNAs are involved in the development of a variety of disorders, such as Alzheimer’s disease,^18^ atherosclerosis^19^ and various tumors.^20^ Therefore, circRNAs have the potential to serve as ideal diagnostic biomarkers. Recent studies have confirmed the presence of large amounts of circRNAs in both peripheral blood and placental tissues of pregnant women and their involvement in PE pathogenesis.^7^,^8^ However, most of these studies have focused on the changes in circRNA expression after the onset of PE. In our nested case-control prospective cohort study, peripheral blood specimens were collected from pregnant women before 20 weeks of gestation and analyzed using microarray technology. We identified 162 differentially expressed circRNAs with a fold of expression of >2.0 that were significantly different in pregnant women with and without PE (P < 0.05). Of them, 30 circRNAs were up-regulated and 132 down-regulated, indicating that differentially expressed circRNAs existed before the onset of PE.

To explore the possible mechanisms of circRNA involvement in PE pathogenesis, our study used bioinformatics analysis to show the host genes of the differentially expressed circRNA mainly participated in protein localization to plasma membrane and cilium, insemination, cellular localization, negative regulation of nitric oxide biosynthetic process, multi-multicellular organism process, etc. In terms of cellular components, the host genes of the differentially expressed circRNA were mainly related to cytoplasmic dynein complex, microtubule cytoskeleton, etc.

In terms of molecular functions, we identified enrichment in dynein light intermediate chain binding, polyubiquitin modification-dependent protein binding, scaffold protein binding, phosphatidylinositol biphosphate kinase activity, peroxisome proliferator activated receptor binding, peroxisome proliferator activated receptor binding, etc. Enrichment analysis of KEGG signaling pathway revealed that differentially expressed circRNAs were involved in the vasopressin-regulated water reabsorption, cGMP-PKG signaling pathway, calcium and other signaling pathway have showed that potential functions of circRNAs may include acting as miRNA sponges or competing with endogenous RNAs (ceRNAs), interacting with RNA-binding proteins (RBPs) as well as regulating gene transcription and mRNA translation^21^ to perform the corresponding biological functions.

The distribution of competing endogenous RNAs (ceRNAs) in the placental tissues of pregnant women with PE was first systematically analyzed by Hu et al.^22^ Additionally, they identified the network relationship between ceRNAs and circRNAs, miRNAs, and long noncoding RNAs (lncRNAs), and used bioinformatics network analysis at different levels to discover that one circRNA, circRNA_0036877, was closely related to the occurrence of PE and could alter trophoblast function. Zhou et al^23^ found that hsa_circ_0002348 could act as an endogenous miR-126-3p sponge, upregulate BAK1 expression, inhibit trophoblast proliferation and promote trophoblast apoptosis, and participate in PE pathogenesis.

In this study, we analyzed the circRNA-miRNA-mRNA regulatory network and found that hsa_circ_0065049 binds miR-17-3p, and hsa_circ_0005567 binds miR-24-3p, which in turn affects target gene mRNA expression. Previous studies have shown that both miR-17-3p and miR-24-3p regulate angiogenesis.^24^,^25^ miR-17-3p also regulates tumor cell proliferation, apoptosis, and cell cycle through p21,^26^ while miR-24-3p affects the growth of PE placental trophoblast cells.^27^ Therefore, we may speculate that hsa_circ_0065049 and hsa_circ_0005567 may be involved in the pathogenesis of PE by affecting the mRNA expression of target genes through the circRNA-miRNA-mRNA regulatory network, which in turn affects the function of related signaling pathways, regulates trophoblast proliferation and apoptosis, and affects the vascular recasting of uterine spiral arteries.

### Limitations:

First, the sample size was small. Blood samples were taken before 20 weeks of gestation and the women were then followed up for pregnancy outcomes. Therefore, only a few samples met the eligibility criteria for inclusion. Further studies with large sample sizes are needed to confirm our results. Secondly, the clinical validation of differentially expressed circRNAs and further investigation of the involvement of circRNAs and interacting miRNAs in the pathogenesis of PE are needed to explain the pathogenesis of PE more clearly and to facilitate the identification of suitable biomarkers for early prediction and preventive targets. In the follow-up research, we aim not only to verify the specific involvement of various circRNAs but also to confirm their specific mechanism of action (including cell and animal experiments).

## CONCLUSIONS

The present study constructed a peripheral blood circRNA expression profile before the onset of PE in pregnant women. We used bioinformatics scores and circRNA-miRNA-mRNA interaction network analysis to explore the potential pathological mechanisms of differentially expressed circRNAs in the process of PE. Our results may provide a new theoretical basis for the early prediction and prevention of PE.

### Authors’ contributions:

**QL** conceived, designed the study and manuscript writing.

**LZ, LX, LJ, JL and JY** collected the data, performed the analysis, critical review.

All authors have read, approved the final manuscript and are responsible for the integrity of the study.
